# On the Frontline: Tracking Ocean Acidification in an Alaskan Shellfish Hatchery

**DOI:** 10.1371/journal.pone.0130384

**Published:** 2015-07-01

**Authors:** Wiley Evans, Jeremy T. Mathis, Jacqueline Ramsay, Jeff Hetrick

**Affiliations:** 1 National Oceanic and Atmospheric Administration, Pacific Marine Environmental Laboratory, Seattle, Washington, United States of America; 2 Ocean Acidification Research Center, School of Fisheries and Ocean Sciences, University of Alaska Fairbanks, Fairbanks, Alaska, United States of America; 3 Alutiiq Pride Shellfish Hatchery, Seward, Alaska, United States of America; The Evergreen State College, UNITED STATES

## Abstract

The invasion of anthropogenic carbon dioxide (CO_2_) into the ocean is shifting the marine carbonate system such that saturation states of calcium carbonate (CaCO_3_) minerals are decreasing, and this is having a detrimental impact on early life stages of select shellfish species. The global, secular decrease in CaCO_3_ saturation states is occurring on top of a backdrop of large natural variability in coastal settings; progressively shifting the envelope of variability and leading to longer and more frequent exposure to adverse conditions. This is a great concern in the State of Alaska, a high-latitude setting vulnerable to rapid changes in the marine carbonate system, where an emerging shellfish industry plans major growth over the coming decades. Currently, the Alutiiq Pride Shellfish Hatchery (APSH) in Seward, Alaska is the only hatchery in the state, and produces many shellfish species with early life stages known to be sensitive to low CaCO_3_ saturation states. Here we present the first land-based OA measurements made in an Alaskan shellfish hatchery, and detail the trends in the saturation state of aragonite (Ω*_arag_*), the more soluble form of CaCO_3_, over a 10-month period in the APSH seawater supply. These data indicate the largest changes are on the seasonal time scale, with extended periods of sub-optimal Ω*_arag_* levels (Ω*_arag_* < 1.5) in winter and autumn associated with elevated water column respiration and short-lived runoff events, respectively. The data pinpoint a 5-month window of reprieve with favorable Ω*_arag_* conditions above the sub-optimal Ω*_arag_* threshold, which under predicted upper-bound CO_2_ emissions trajectories is estimated to close by 2040. To date, many species in production at APSH remain untested in their response to OA, and the data presented here establish the current conditions at APSH as well as provide a framework for hatchery-based measurements in Alaska. The current and expected conditions seen at APSH are essential to consider for this developing Alaskan industry.

## Introduction

A total of nearly 155 Pg (1 Pg = 10^15^ g) of carbon have been taken by the ocean due to rising atmospheric carbon dioxide (CO_2_) levels that result largely from anthropogenic fossil fuel emissions [1]. Ocean CO_2_ uptake increases the concentration of hydrogen ions (H^+^; lowers seawater pH) while also decreasing carbonate ion (CO_3_^2-^) levels; reactions that collectively have been termed ocean acidification (OA) [2–6]. Decreasing CO_3_^2-^ has the effect of reducing the saturation state of calcium carbonate (CaCO_3_) by the equation: Ω_*phase*_ = [Ca^2+^][CO_3_^2-^]/K_sp_; where Ω_*phase*_ is the saturation state for typically either aragonite or calcite phases of CaCO_3_, Ca^2+^ is the calcium concentration, and K_sp_ is the phase specific solubility product. Of these reactions associated with OA, a decrease in Ω_*phase*_ is a critical alteration in carbonate chemistry affecting rates of calcification and shellfish growth [7, 8]. For the aragonite phase of CaCO_3_, Ω_*arag*_ values where sub-lethal sensitivity first become evident for a number of bivalve larvae range between 1.2 and 2.0 [7]. At Ω_*arag*_ < 1.0, thermodynamics dictate aragonite will actively dissolve in seawater [9]. An unfavorable condition for aragonite precipitation is a considerable stressor for early life stages of select shellfish species that can ultimately lead to mortality [7, 8]. Due to the critical nature of this carbonate system parameter in dictating shellfish production, it is imperative for aquaculture facilities to accurately track Ω_*arag*_ in their seawater supplies. In the State of Alaska, having this capacity is essential for an emerging shellfish industry in the midst of a shifting biogeochemical seascape [10].

Over the time scale of a year, ocean uptake of anthropogenic CO_2_ results in small incremental chemical changes. On decadal time scales, these changes can shift baselines in marine carbonate chemistry with potentially significant ecosystem level impacts [11–13]. This is especially the case in high-latitude settings where anthropogenically-driven reductions in Ω_*arag*_ are expected to occur ahead of changing conditions in warmer locales [14, 15]. The risk associated with such wholesale biogeochemical shifts in the marine environment will potentially have far-reaching effects beyond the ecosystems themselves and the dependence of coastal communities on ocean goods and services [10, 15]. Aquaculture hatcheries have the capacity to be important contributors to food security in support of growing populations by ingesting seawater from adjacent coastal settings in order to produce marketable marine species. As such, hatcheries are on the frontline both in terms of being one of the first industries impacted by OA [8, 11, 15, 16] and in their need to track and cope with the changing ocean biogeochemical conditions. It is important to note that in vulnerable coastal settings, secular changes in CO_2_ are occurring in conjunction with large, in many cases understudied, carbonate system variability; demonstrated, for instance, by data collected on the United States (U.S.) west coast (Oregon) continental shelf over multiple years [17]. As shown by Harris et al. [17], large carbonate system variability does not diminish the importance of an increasing trend. Effectively, the gradual human-induced changes in Ω_*arag*_ shift the envelope of variability such that organisms experience more adverse conditions longer and more often than they would otherwise [17–19].

Pacific oyster, *Crassostrea gigas*, is a cultured species that has been described as a “canary in the coalmine” for being an organism highly impacted by reduced Ω_*arag*_ levels in the coastal zone [8, 16, 20]. Pacific oyster production failures in U.S. Pacific Northwest (PNW) shellfish hatcheries between 2005 and 2009 resulted from the ingestion of low- Ω_*arag*_, high-pCO_2_ water, which is naturally upwelled along the coast, into shore-side hatchery facilities. As detailed by Waldbusser et al. [21], adverse Ω_*arag*_ conditions dually impact larval *Crassostrea gigas* during D-hinge (prodissoconch I) shell formation by: (1) exposing calcification surfaces until completion of the D-hinge shell, and (2) increasing kinetic demands that then impacts the energy budget already limited by endogenous resources. The combination of these factors explains the sensitivity to Ω_*arag*_ > 1 when aragonite is thermodynamically favored to precipitate. The additional anthropogenic CO_2_ signal in upwelled water along the PNW is small relative to natural CO_2_ levels in upwelling source waters [22], however large enough to alter the natural envelop of variability in these systems and impart a significant stress that resulted in a near collapse in a $270 Million industry for the State of Washington [23]. The hatchery production failures served as a vital catalyst in two ways [16]: (1) by unifying stakeholders and scientists in establishing robust monitoring techniques to track Ω_*arag*_ in hatchery intake water [8], and (2) by instigating state and federal government action to fund scientific activities needed to catalog trends in coastal manifestations of OA along with their impacts on marine ecosystems and the economies they support. To this end, data are presently streaming in near real-time from a number hatchery facilities along the Pacific coast of North America [24], and guidelines have been developed to aid stakeholders in making OA related land-based measurements [25].

**Fig 1:**
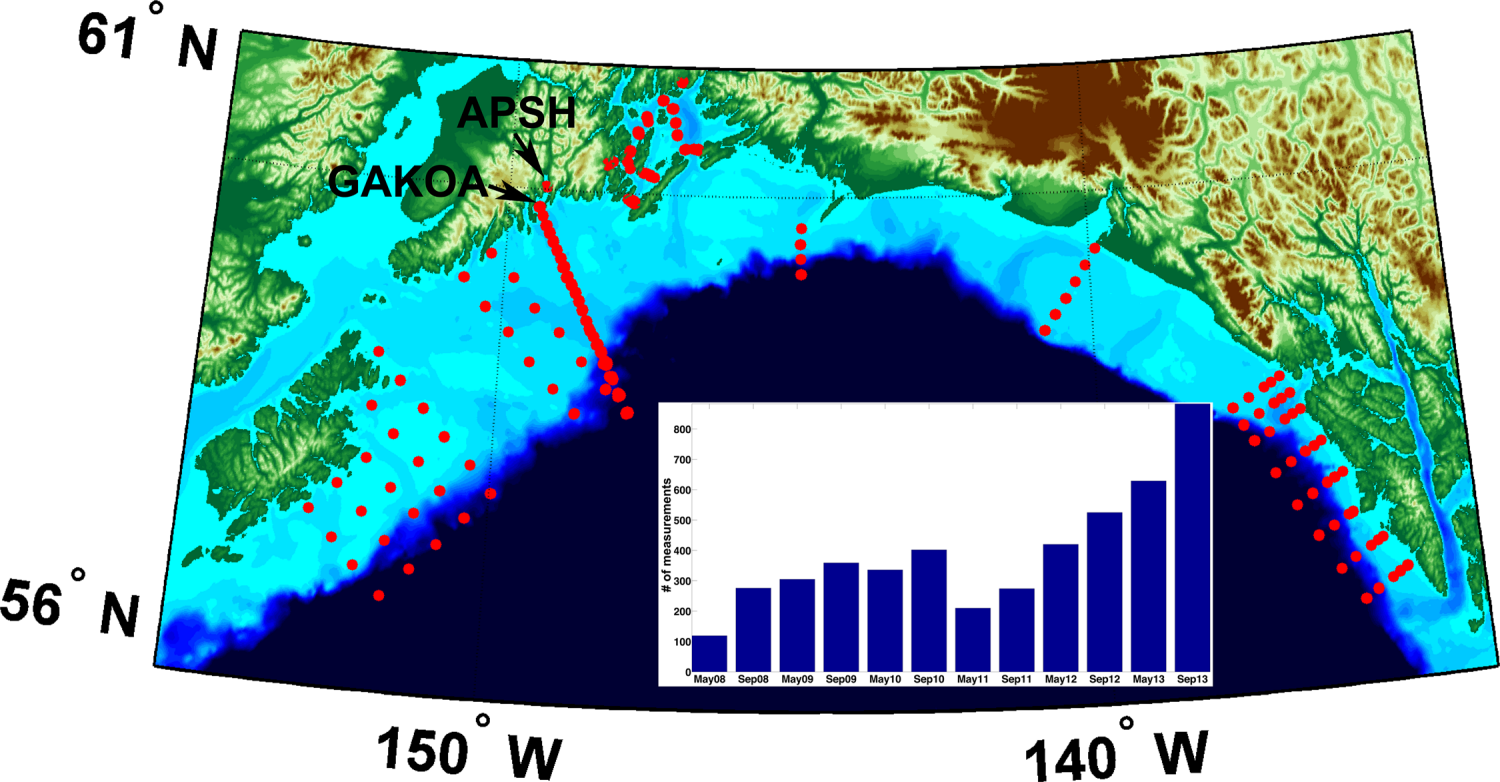
Map showing the location of Alutiiq Pride Shellfish Hatchery (APSH) in Resurrection Bay, the position of the Gulf of Alaska OA (GAKOA) mooring at the mouth of Resurrection Bay. Red dots are locations of discrete total alkalinity (TA) and salinity measurements, with the number of measurements made during cruises in May and September from 2008 through 2013 shown in the insert.

In this contribution, we report on land-based determinations of Ω_*arag*_ from seawater entering an Alaskan hatchery facility in the northern Gulf of Alaska (GOA): the Alutiiq Pride Shellfish Hatchery (APSH) [26]. APSH is located in Seward, Alaska on the shore of Resurrection Bay (Fig 1) and is currently the only shellfish hatchery in the state. This is expected to change in the near future, as the Alaska Fisheries Development Foundation, Inc. currently seeks to pass an Alaska Mariculture Initiative with the aim of growing the industry to a $1 billion level in 30 years [27]. APSH is the primary producer of Blue and Red Alaskan King crab, *Paralithodes platypus* and *Paralithodes camtschaticus*, respectively. The latter crab species has been shown to be sensitive to low Ω_*arag*_ levels [28]. APSH also produces other animals that have either previously shown sensitivity to reduced Ω_*arag*_ (i.e. Pacific oyster [7, 8]) or are believed to be vulnerable during early life stages (*Panopea generosa*, *Crassadoma gigantea*, *Leukoma staminea*). Prior to the data presented here, it was unclear whether APSH had been experiencing threatening levels of Ω_*arag*_ in their seawater supply. APSH is unique owing to its close proximity to an area of intense OA-related sampling from central Resurrection Bay to over the adjacent continental shelf [29]. Full water column measurements have been made in this area every May and September since 2008, and the GOA OA (GAKOA) mooring has been in place at the mouth of Resurrection Bay since 2011(Fig 1) [30]. Using high-speed measurements of the seawater entering the hatchery and a compiled dataset from the adjacent coastal ocean, trends in Ω_*arag*_ were resolved over a 10-month period and projections were made for the changing conditions at this integral site for the state of Alaska’s growing shellfish industry.

## Materials and Methods

From October 5, 2013 to August 5, 2014, temperature, salinity and CO_2_ partial pressure (pCO_2_) data were collected from the seawater supply flowing into APSH. This seawater supply was provided to the hatchery by the University of Alaska D. W. Hood Laboratory at the Seward Marine Center [31]. Untreated seawater was pumped to the facilities from an intake pipe extending to 75 m depth and 91 m from the laboratory into Resurrection Bay. Two seawater pumps provided flow at ~200 gallons per minute (gpm), and a tangential split off the main supply line provided seawater at ~1 gpm to the hydrographic and chemical sensors positioned in the preparation room of the D.W. Hood Laboratory. Temperature and salinity (calculated from conductivity) were measured with a Sea-Bird Electronics SBE 45 MicroTSG thermosalinograph [32], with salinity reported in this paper using the Practical Salinity Scale (PSS-78, dimensionless).

Seawater pCO_2_ data were calculated from corrected measurements of CO_2_ mixing ratio (xCO_2_) made using a Sunburst Sensors SuperCO_2_ System [33] following protocols recommended by Pierrot et al. [34] with the system theory and calculations described in detail elsewhere [35–38]. Seawater continuously flowed first through the thermosalinograph and then through two (primary and secondary) showerhead equilibrators [34, 39]. The primary showerhead equilibrator supplied equilibrated carrier gas (marine air) to a non-dispersive infrared gas analyzer (LI-COR LI840A CO_2_/H_2_O) housed within the SuperCO_2_ system’s electronics box at a rate of ~50 ml min^-1^. Unaltered marine air was drawn from 0.25 inch polyethylene tubing that connected a vented water trap positioned outside of the D.W. Hood Laboratory to the secondary equilibrator. Marine air was pre-equilibrated in the secondary equilibrator, and then plumbed to the primary equilibrator as a make-up air supply to replace equilibrated carrier gas provided to the LI-COR. Pressure and temperature were continuously measured in the primary equilibrator using a Honeywell ASCX Microstructure Pressure Sensor and a Minco Fast Response RTD, respectively. Equilibrated carrier gas, three standard gases of known mixing ratio (148, 448 and 748 ppm; Scott-Marin, Inc.), and unaltered marine air were all plumbed to provide gas flow to the SuperCO_2_ system’s electronics box. The SuperCO_2_ system was controlled using National Instruments LabVIEW software run on an HP laptop computer. The software controls data acquisition from the thermosalinograph, the pressure and temperature sensors, and the LI-COR; while also controlling Valco Instruments Co. Inc. (VICI) multi-port actuators that cycle between the gas streams plumbed to the electronics box. None of the gas streams were dried prior to analysis, and all measurements were made at 0.5 Hz. The prescribed measurement scheme controlled by the software was to supply equilibrated carrier gas from the primary equilibrator to the LI-COR continuously for 240 minutes, then cycle the actuators to consecutively allow the three standard gas streams and unaltered marine air to be measured for 90 s at 100 ml min^-1^ before returning to sample the carrier gas equilibrated with seawater xCO_2_. From each sequence of standard gas measurements, the final 20 s of data in the 90 s interval before the actuator changed position were used to construct calibration functions that were then interpolated in time between standard gas sequences. These functions were then used to calibrate the xCO_2_ measurements of seawater equilibrated carrier gas, with an adjustment on the order of 1%. Atmospheric xCO_2_ measurements made by the SuperCO_2_ system were not used in this analysis, as these data do not enter in to calculations of Ω_*arag*_. Annual mean atmospheric pCO_2_ was calculated from the well-resolved data collected on the GAKOA mooring. This annual mean atmospheric pCO_2_ value was then used in anthropogenic CO_2_ calculations. Corrected seawater xCO_2_ was subsequently adjusted for under-pressurization in the primary showerhead equilibrator using the ratio of equilibrator to vented LI-COR cell pressure, and then converted to pCO_2_ using atmospheric pressure measured by the LI-COR. The 2-s seawater pCO_2_, temperature and salinity data were quality controlled, and then bin-averaged in 5-min interval bins.

Taking advantage of the expansive and growing dataset of paired total alkalinity (TA) and salinity measurements made throughout the northern GOA coastal ocean (Fig 1; [29]), these data were compiled and used to construct a TA–salinity relationship. The compiled data were collected as part of the growing suite of carbonate system and hydrographic measurements made from oceanographic cruises that have occurred every May and September since 2008. Briefly, seawater samples were collected at specific stations and depths using a rosette equipped with 5-l Niskin bottles and a Sea-Bird Electronics 911Plus conductivity-temperature-depth (CTD) profiler. Seawater was drawn from Niskin bottles into clean 250 ml Pyrex glass reagent bottles using established sampling techniques [40], and treated with 200 μl of saturated HgCl_2_ solution to halt biological activity in the samples. TA was measured in the fixed seawater samples at the University of Alaska Fairbanks (UAF) Ocean Acidification Research Center (OARC) using a VINDTA 3C [41]. Seawater certified reference materials (provided by A. G. Dickson, Scripps Institute of Oceanography) were analyzed before samples were processed to ensure measurements were accurate to within 0.1% (2 μmol kg^-1^). A robust linear regression was computed between the TA and salinity data using MathWorks MATLAB software. A small number of TA validation measurements (n = 23) were collected at APSH to verify the credibility of this linear relationship (S1 Table). 5-min binned seawater pCO_2_ data, TA derived using the TA–salinity relationship, temperature and salinity were input into a customized MathWorks MATLAB version of CO2SYS [42] for carbonate system calculations. pH on the total hydrogen ion scale (pH_T_), CO_3_^2-^ and Ω_*arag*_ were computed using the equilibrium constants for the dissociation of carbonic acid from Millero [43]. Ca^2+^ concentrations were calculated from salinity using the relationship from Riley and Tongudai [44]. For estimating the contribution of anthropogenic CO_2_ in setting Ω_*arag*_ variability at APSH, the mean atmospheric pCO_2_ from GAKOA (398 ± 6.7 μatm) was used to calculate the sea-air difference in pCO_2_ (∆pCO_2_). While the term “sub-lethal” has been used in the literature to describe the impact of Ω_*arag*_ levels < 1.5 on select larval shellfish species [7, 8, 15], to date, there has been no evidence of die-offs directly linked to Ω_*arag*_ conditions at APSH. Here, we consider Ω_*arag*_ values ≤ 1.5 to be sub-optimal growth conditions for many larval species produced by APSH (e.g. *Crassostrea gigas*); Ω_*arag*_ equal to 1.5 marks the threshold value below which increased stress and vulnerability is expected [7, 15].

## Results and Discussion

**Fig 2:**
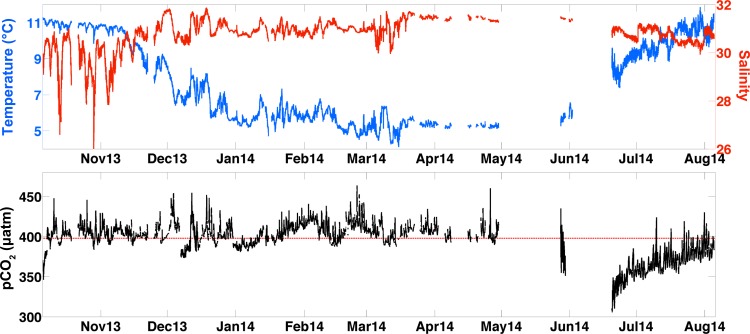
Ten months of data collected from intake water entering the Alutiiq Pride Shellfish Hatchery (APSH). The top panel is temperature (°C; blue) and salinity (red), and the bottom panel is seawater pCO_2_ (μatm) with the red dashed horizontal line as the mean atmospheric concentration (398 ± 6.7 μatm).

**Fig 3:**
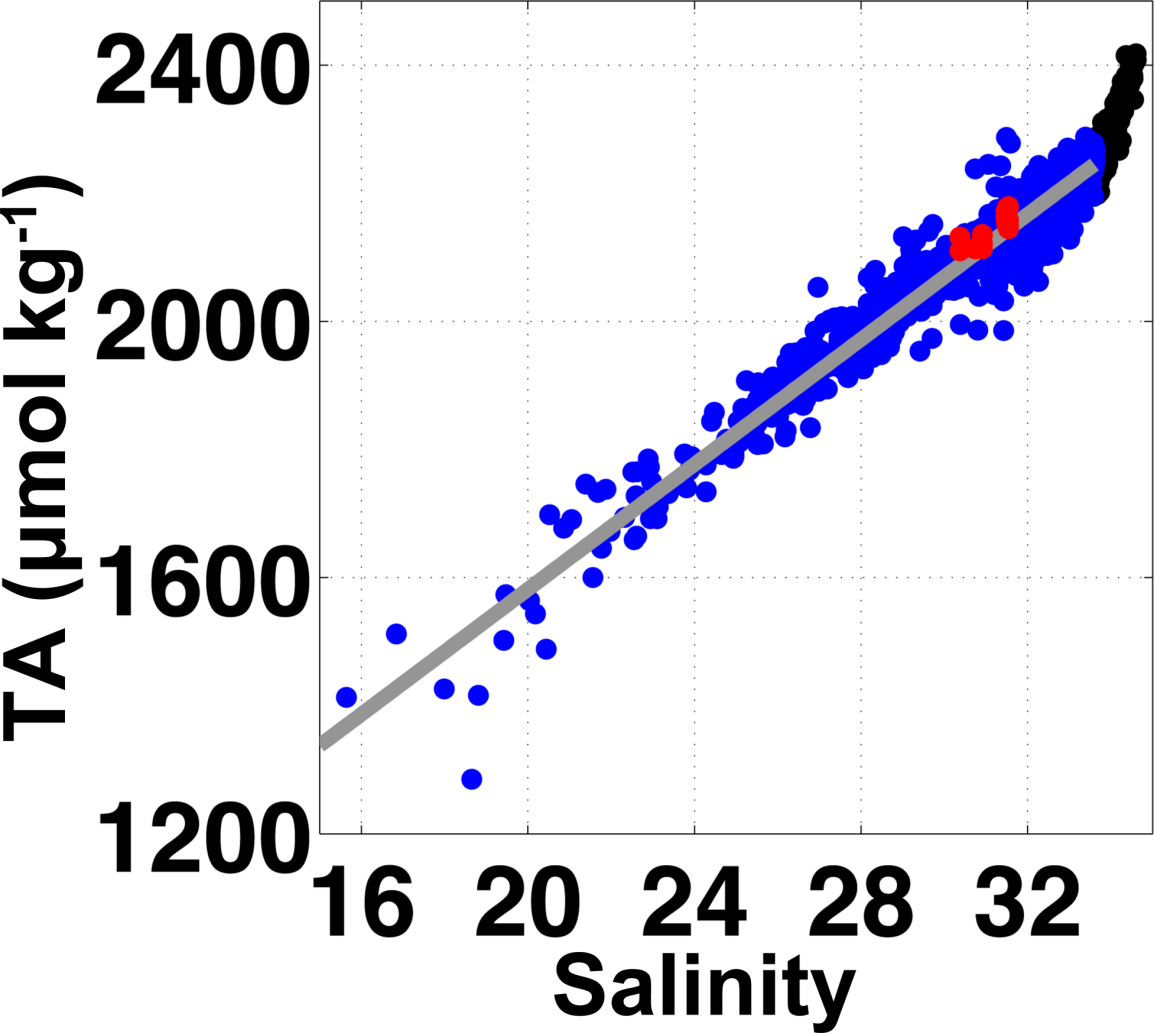
Relationship between total alkalinity (TA; μmol kg^-1^) and salinity in the northern Gulf of Alaska calculated using the data described in Fig 1. The linear fit (gray line) for these data is: TA = 48.7709*S + 606.23 μmol kg^**-1**^ (r^**2**^ = 0.94, root mean square error = 17.21 μmol kg^**-1**^). This fit was calculated using the MathWorks MATLAB robust linear regression algorithm with only salinity data < 33.6 (blue dots). Measurements above this salinity range are deep samples collected over the outer continental shelf that have a steeper TA-salinity relationship (black dots). Twenty-three validation TA measurements (S1 Table) were made and are shown here as red dots.

**Fig 4:**
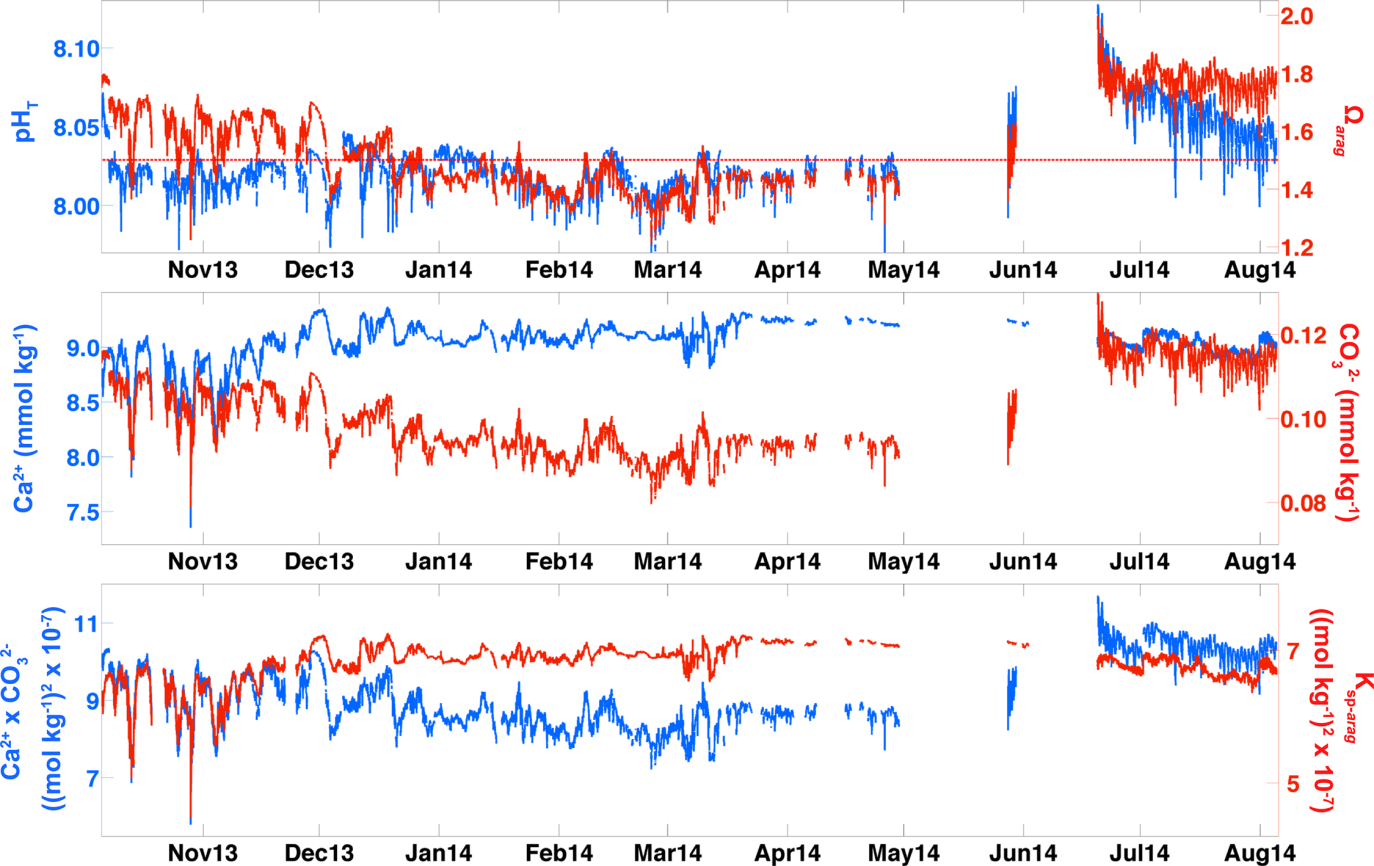
Top panel is pH on the total hydrogen ion scale (pH_T_) and the saturation state of the aragonite phase of CaCO_3_ (Ω_*arag*_). The dashed red line is the sub-optimal 1.5 Ω_*arag*_ threshold. Subsequent panels are a decomposition of Ω_*arag*_ components: middle panel is calcium (Ca^**2+**^) and carbonate (CO_3_^**2-**^) concentrations (mmol kg^**-1**^), and bottom panel is Ca^**2+**^ x CO_3_^**2-**^ ((mol kg^**-1**^)^**2**^ x 10^**−7**^) and the solubility product for aragonite (K_sp-arag_; (mol kg^**-1**^)^**2**^ x 10^**−7**^).

Presented here are the first Ω_*arag*_ values from seawater entering an Alaskan shellfish hatchery, and the data indicate unique characteristics setting conditions at APSH apart from those at other hatcheries to the south along the U.S. Pacific coast within the California Current System [8]. In source waters to APSH, temperature and salinity underwent only modest changes throughout the period of observation, with temperature ranging from 5 to 11°C and salinity varying between 26 and 32 (Fig 2). pCO_2_ spanned 305 to 463 μatm, and was relatively confined in comparison to the dynamic range seen in surface water measured by the GAKOA buoy at the mouth of Resurrection Bay [45]. At this nearby surface site, only limited pCO_2_ data above atmospheric levels have been observed to occur between February and March [30]. The most prolonged periods of high pCO_2_ levels at APSH were from January to April (Fig 2). Note that record gaps in April, May and June ranged from 2 to 20 days, and were caused by either electrical power outages in Seward or technical issues associated with the SuperCO_2_ system. The TA–salinity relationship built from compiled GOA data [29] was robust (Fig 3) as indicated by a high r^2^ value (0.94), low root mean square error (RMSE; 17.21 μmol kg^-1^), and close agreement with our validation samples (S1 Table). Ω_*arag*_, calculated using TA from the TA–salinity relationship with the pCO_2_ data, showed a smaller dynamic range of relative to the measurements made within PNW hatcheries [8], with only a 0.8 unit oscillation about the 1.5 Ω_*arag*_ threshold over the 10-month record (Fig 4). The largest abrupt changes in Ω_*arag*_ were seen during autumn runoff events (0.5 unit), whereas during July and August the near 40-μtam diurnal signal in pCO_2_ (Fig 2) amounted to only 0.1 unit daily changes in Ω_*arag*_ (Fig 4). This small variability in Ω_*arag*_ is greater than the at most 0.06 unit error in the Ω_*arag*_ calculation estimated by propagating the RMSE value from the TA–salinity relationship with the error in the pCO_2_ data (2.1 μatm; dominated by error in the LI-COR LI840A calibration functions). Short-term diurnal variability in the Alaskan hatchery is minimal in comparison to the extremes seen at the Whiskey Creak Hatchery (WCH) in Netarts Bay, Oregon where Ω_*arag*_ can vary by more than a whole unit over a day [8]. Ω_*arag*_ varies more on seasonal time scales at APSH, with highest values in July and August and low sub-optimal levels persisting from January to April. October through December is a transition period to the prolonged sub-optimal conditions in winter, which is punctuated by abrupt short-lived decreases in Ω_*arag*_. Assuming that May and June also are transition months to the high summer Ω_*arag*_ levels, and that conditions in September are similar to those of August, this implies a 5-month window (May through September) of favorable Ω_*arag*_ conditions at APSH. During these 5 months, prolonged Ω_*arag*_ levels above the sub-optimal 1.5 Ω_*arag*_ threshold should alleviate this as a stressor for larval shellfish production, whereas during the other months of the year this may not be the case.

Over the 10-month period, pH_T_ ranged from 7.96 to 8.13 and averaged 8.03 ± 0.02. Average Ω_*arag*_ was near the sub-optimal threshold at 1.55 ± 0.15, with levels varying from 1.2 to 2.0. pH_T_ and Ω_*arag*_ closely tracked each other for only 6 of the 10 months of observations (Fig 4). From January to June, pH_T_ and Ω_*arag*_ were tightly coupled, with Ω_*arag*_ largely near or below the 1.5 Ω_*arag*_ threshold. pH_T_ and Ω_*arag*_ became decoupled in July, August, October and November, with pH_T_ depressed while Ω_*arag*_ persisted above the sub-optimal Ω_*arag*_ threshold, except during short-lived periods of abrupt decreases in Ω_*arag*_. Maximal error in the pH_T_ calculations estimated by propagating pCO_2_ and TA–salinity errors was 0.01 units, well below the 0.07 unit drop in pH_T_ during the pH_T_− Ω_*arag*_. The pH_T_ and Ω_*arag*_ decoupling was opposite in direction to what was observed in surface water impacted by glacial melt within nearby Prince William Sound [46], and highlights the absolute requirement to track two concurrent carbonate system parameters in order to constrain the carbonate system in coastal settings [8, 25, 47]. In this case, the decoupling was entirely temperature driven. As eloquently described by Takahashi et al. [48], the equilibrium constants for the dissociation of carbonic acid, and the K_sp-*arag*_, are temperature and salinity dependent, with the second equilibrium constant for the dissociation of carbonic acid increasing 1.5x faster with increasing temperature than the first. This inequality in the temperature response between the two equilibrium constants drives an adjustment within the carbonate system to increase carbonic acid and CO_3_^2-^ while decreasing bicarbonate ion in order to maintain charge and mass balances. Carbonic acid equals the product of CO_2_ solubility and pCO_2_, and the required increase in pCO_2_ drives an increase in H^+^ lowering pH_T_. In addition to the increase in CO_3_^2-^ caused by the temperature-driven shift within the carbonate system, K_sp-*arag*_ decreases with increasing temperature and the sum of these two effects raises Ω_*arag*_ levels. For the months of July, August, October and November at APSH, utilizing pH alone as an indication of Ω_*arag*_ would produce misleading and inaccurate results.

**Fig 5:**
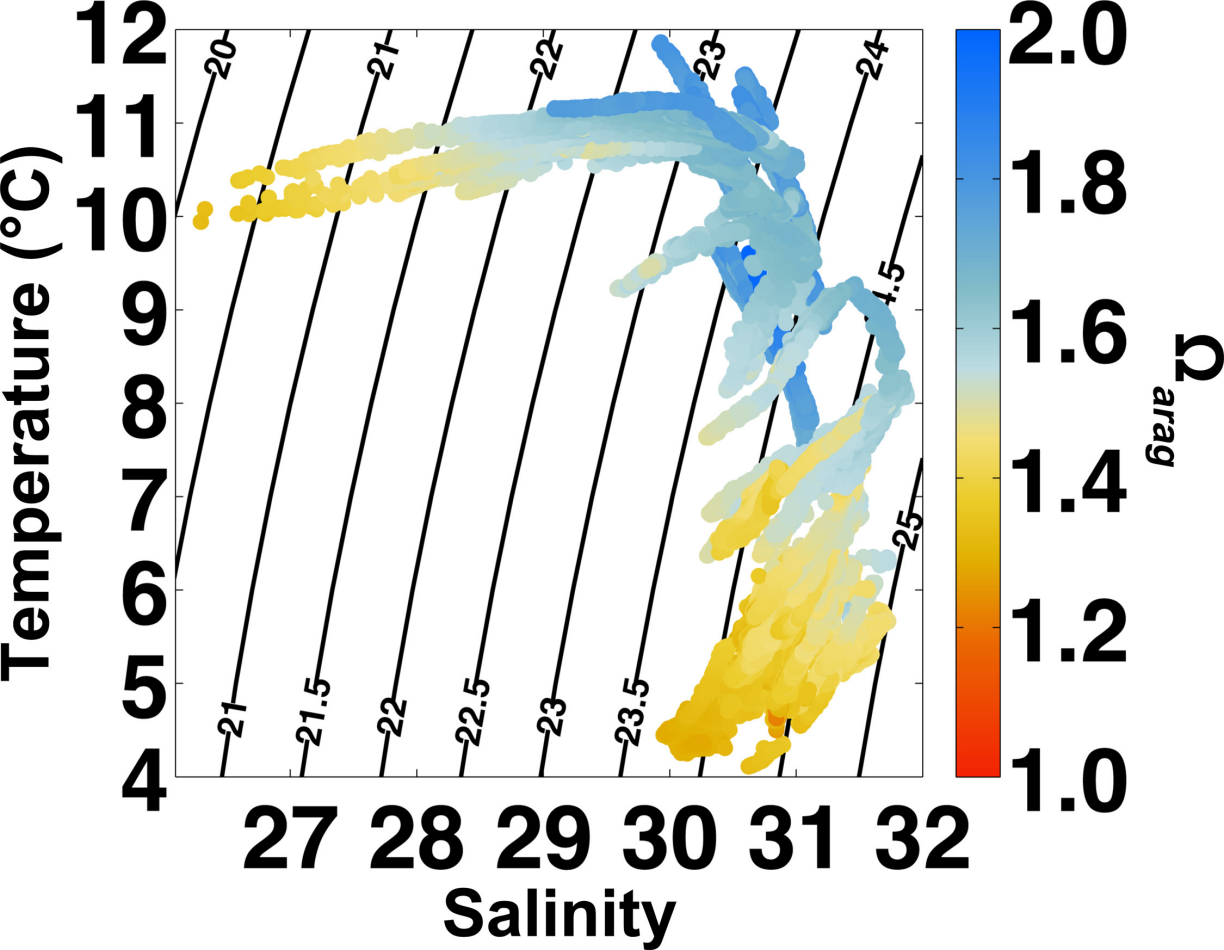
Temperature (°C)–salinity diagram with contours of seawater potential density anomaly (σ_t_) and Ω_*arag*_ as colored dots. Note the two areas of sub-optimal Ω_*arag*_ (Ω_*arag*_ < 1.5; warm colors) at the lowest salinities (<29) and coldest temperatures (< 7°C).

Using output Ca^2+^, CO_3_^2-^ and K_sp-*arag*_ data from CO2SYS, the Ω_*arag*_ record was decomposed into its constituents in order to pinpoint the drivers of sub-optimal Ω_*arag*_ levels that occurred during months outside of the window of favorable Ω_*arag*_ conditions (Fig 4). Ca^2+^ in the seawater supply to the hatchery ranged between 7.5 and 9.5 mmol kg^-1^, dropping to lowest values only during brief periods when low salinity surface water was mixed vertically to the depth of the intake in October and November (Figs 2 and 4). CO_3_^2-^ levels vary over a narrower range, between 0.08 and 0.13 mmol kg^-1^, and were highest during the period of sustained low pCO_2_ and warm water temperatures during July and August (Figs 2 and 4). The dynamic range of CO_3_^2-^, 51 μmol kg^-1^, was 13x the error of 3.9 μmol kg^-1^ calculating by propagating pCO_2_ and TA–salinity errors. During winter months, Ca^2+^ and K_sp-*arag*_ were largely stable and near 9 mmol kg^-1^ and 7 (mol kg^-1^)^2^ x 10^−7^, respectively. The decline in Ω_*arag*_ over this period resulted from the steady decrease in CO_3_^2-^ to a minimum in February and March (Fig 4), consistent with the period of sustained pCO_2_ above atmospheric levels (Fig 2) and indicating a respiration-driven depression in Ω_*arag*_. During October and November, CO_3_^2-^ decreases abruptly (Fig 4) along with short-lived increases in pCO_2_ above atmospheric levels (Fig 2). During this time, CO_3_^2-^, Ca^2+^ and K_sp-*arag*_ all decrease precipitously during the episodic low salinity events (Figs 2 and 4). Low Ω_*arag*_ conditions during these autumn months are triggered by runoff events and match the intensity but not the length of exposure of the respiration-driven decline in Ω_*arag*_ seen in winter (Fig 4). In temperature-salinity space, the two differing water masses responsibly for these trends in Ω_*arag*_ become readily apparent, and this provides a framework for avoidance of sub-optimal Ω_*arag*_ levels (Fig 5). Temperature-salinity data collected over the 10-month period show a two-pronged distribution, with sub-optimal Ω_*arag*_ levels seen in water masses with the lowest salinities (<29) and coldest temperatures (< 7°C). Both of these water mass types have high pCO_2_ with respect to the atmosphere (Fig 2), and these temperature–salinity–pCO_2_ characteristics set up a diagnostic for APSH to respond to adverse Ω_*arag*_ conditions in the absence of real-time Ω_*arag*_ calculations.

**Fig 6:**
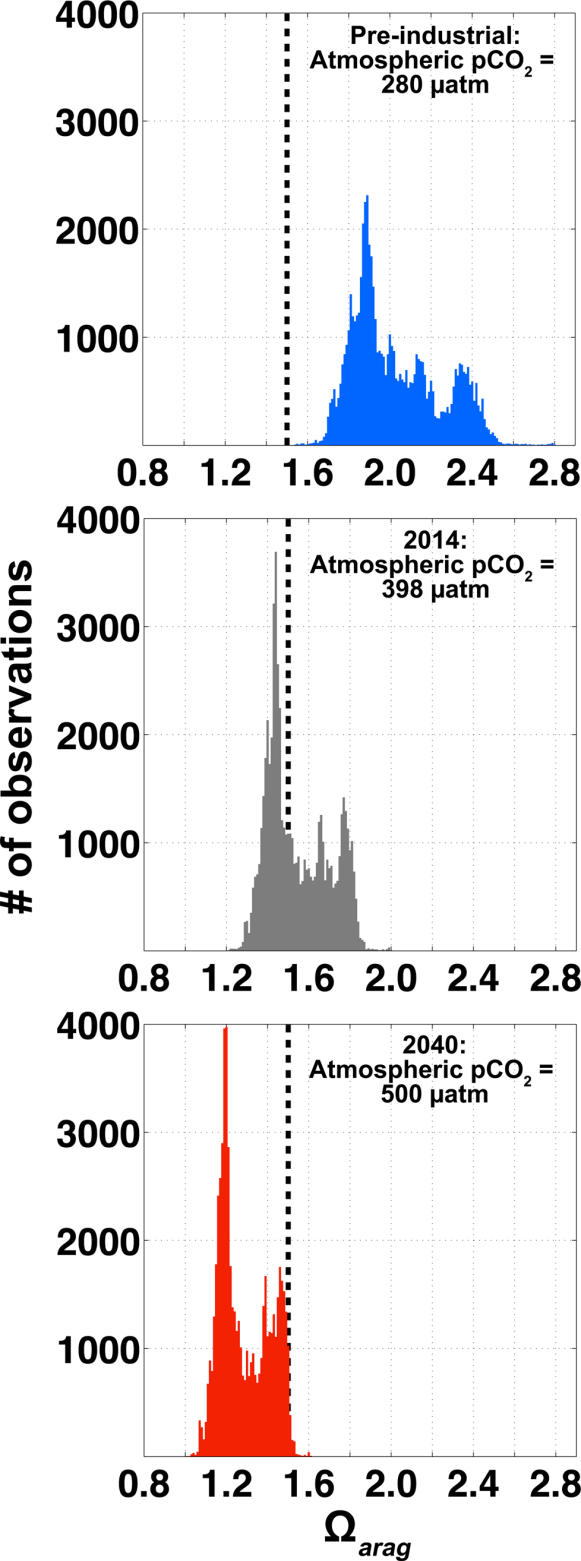
Bar graphs showing histograms of Ω_*arag*_ observations at APSH. The center panel is the data shown in Fig 4 with 2014 atmospheric pCO_2_ levels (398 μatm; gray). Top and bottom panels are Ω_*arag*_ computed using total CO_2_ (TCO_2_) adjusted to atmospheric pCO_2_ levels of 280 (blue) and 500 (red) μatm, respectively, following the approach of Harris et al. [17] by assuming sea-air CO_2_ disequilibria and the processes that determine TA, temperature and salinity variability are constant in time. The vertical dashed black line in all panels is the sub-optimal 1.5 Ω_*arag*_ threshold where some early life stages of marine bivalves become stressed [7, 8]. An atmospheric pCO_2_ of 500 is expected by 2040 if the IPCC AR5 RCP 8.5 emissions trajectory is realized.

The 5-month window of favorable Ω_*arag*_ conditions at APSH will shrink in the future under the continued increase in anthropogenic CO_2_ emissions. Using the approach of Harris et al. [17], we can subtract the anthropogenic CO_2_ signal from the APSH pCO_2_ data by making the assumptions that the sea-air CO_2_ disequilibrium is roughly constant as are the processes shaping the distributions of TA, salinity and temperature. In this same way, we can also add additional anthropogenic CO_2_ to pinpoint when the window of favorable growth conditions may close. Target atmospheric pCO_2_ levels were added to APSH ∆pCO_2_ data computed using the mean atmospheric pCO_2_ from GAKOA (Figs 1 and 2) with the result being an adjusted seawater pCO_2_ record. Ω_*arag*_ at the target atmospheric pCO_2_ levels were calculated with CO2SYS using the adjusted seawater pCO_2_, TA, salinity and temperature data. Under a pre-industrial atmospheric pCO_2_ level (pCO_2_ = 280 μatm), the window for favorable Ω_*arag*_ conditions is open the entire year (Fig 6); none of the estimated Ω_*arag*_ levels fell below the 1.5 Ω_*arag*_ threshold. Note that the average difference between total CO_2_ calculated using both unadjusted seawater pCO_2_ and adjusted seawater pCO_2_ to a pre-industrial atmosphere with TA, salinity and temperature data was 52.4 μmol kg^-1^. This was in close agreement with an independent estimate of the total CO_2_ addition from anthropogenic CO_2_ uptake on density surfaces < 26.9 kg m^-3^ in the North Pacific until 2006 and scaled to 2014 using a growth rate of 1.62 μmol kg^-1^ yr^-1^ (47.96 μmol kg^-1^) [49]. Atmospheric pCO_2_ concentrations were then iteratively increased beyond the mean 2014 atmospheric level to estimate when the window of favorable Ω_*arag*_ conditions may close. The atmospheric pCO_2_ at which this occurred was 500 μatm (Fig 6). Under the Intergovernmental Panel on Climate Change (IPCC) representative concentration pathway (RCP) emissions scenario 8.5, this atmospheric pCO_2_ threshold will be reached by approximately 2040 [50]. Ω_*arag*_ levels at APSH in 2040 would still remain above the thermodynamic threshold for aragonite dissolution (Ω_*arag*_ = 1; Fig 6), but clearly seawater manipulation strategies will need to be in place by this time in order to facilitate conditions favorable for larval shellfish production.

## Conclusions

The window of favorable Ω_*arag*_ conditions in source waters to APSH is gradually closing, and this biogeochemical shift is consistent across the coastal Gulf of Alaska [10, 15, 51]. In 3 decades, sub-optimal Ω_*arag*_ levels will be a constant condition for this premier Alaskan hatchery (Fig 6). Manipulation strategies will need to be invoked to alter the carbonate chemistry of the seawater supply, or alternatively draw seawater from a shallower depth at the risk of experiencing a higher frequency of low Ω_*arag*_ runoff events. Inherently this implies a decreased volume in the water column of Resurrection Bay containing Ω_*arag*_ above the sub-optimal threshold, and this has implications for natural populations of vulnerable shellfish species in the region. A limited number of studies examining biological impacts due to shifting carbonate chemistry in Alaskan coastal waters exist [28], and additional studies are essential for detailing the severity of these changes for natural and cultured assemblages. Most of the species currently in production at APSH remain untested in their response to OA. The data presented here establish the current conditions experienced by APSH, and provide a framework for hatchery-based measurements in Alaska. For Alaska’s growing shellfish aquaculture industry to reach its target growth and be successful in the shifting biogeochemical climate of the coastal ocean, the implementation of robust measures for tracking Ω_*arag*_ are key, as are strong partnerships between stakeholders and scientists. State and federal government provision of OA-related scientific research that directly supports stakeholders such as the Alaskan shellfish industry is an excellent model for simultaneously backing industry while progressing scientific initiatives.

## Supporting Information

S1 Table Validation measurements (n = 23) of temperature, salinity and total alkalinity collected in the hatchery during the 10-month period of observation. (EPS)
